# *Trans*-**β**-Caryophyllene: An Effective Antileishmanial Compound Found in Commercial Copaiba Oil (*Copaifera* spp.)

**DOI:** 10.1155/2013/761323

**Published:** 2013-06-22

**Authors:** Deivid C. Soares, Nathalya A. Portella, Mônica Freiman de S. Ramos, Antonio C. Siani, Elvira M. Saraiva

**Affiliations:** ^1^Laboratório de Imunobiologia das Leishmanioses, Instituto de Microbiologia Paulo de Góes, Universidade Federal do Rio de Janeiro, Avenida Carlos Chagas Filho 373, Bloco D, Sala D1-44, Ilha do Fundão, 21941-902 Rio de Janeiro, RJ, Brazil; ^2^Faculdade de Farmácia, Centro de Ciências da Saúde, Bloco K, 2° Andar, Universidade Federal do Rio de Janeiro, Cidade Universitária, Ilha do Fundão, 21941-902 Rio de Janeiro, RJ, Brazil; ^3^Departamento de Produtos Naturais, Instituto de Tecnologia em Fármacos, Fundação Oswaldo Cruz, Rua Sizenando Nabuco 100, Manguinhos, 21041-250 Rio de Janeiro, RJ, Brazil

## Abstract

This study investigated the leishmanicidal activity against *Leishmania amazonensis* of four commercial oils from *Copaifera* spp. named as C1, C2, C3, and C4, the sesquiterpene and diterpene pools obtained from distilling C4, and isolated **β**-caryophyllene (CAR). Copaiba oils chemical compositions were analyzed by gas chromatography and correlated with biological activities. Diterpenes-rich oils C2 and C3 showed antipromastigote activity. Sesquiterpenes-rich C1 and C4, and isolated CAR presented a dose-dependent activity against intracellular amastigotes, with IC_50s_ of 2.9 **µ**g/mL, 2.3 **µ**g/mL, and 1.3 **µ**g/mL (6.4 **µ**M), respectively. Based on the highest antiamastigote activity and the low toxicity to the host cells, C4 was steamdistillated to separate pools of sesquiterpenes and diterpenes. Both pools were less active against *L. amazonensis* and more toxic for the macrophages than the whole C4 oil. The leishmanicidal activity of C3 and C4 oils, as well as C4 fractions and CAR, appears to be independent of nitric oxide production by macrophages. This study pointed out **β**-caryophyllene as an effective antileishmanial compound and also to its role as potential chemical marker in copaiba oils or fractions derived thereof, aiming further development of this rainforest raw material for leishmaniasis therapy.

## 1. Introduction

Leishmaniasis comprises a group of neglected tropical diseases caused by the protozoan parasite *Leishmania*. It is estimated that 12 million people are infected worldwide and every year 0.9–1.6 million people develop symptomatic disease [1, 2]. The first-choice treatment for leishmaniasis still relies on pentavalent antimonials, and amphotericin B or pentamidine can be used as alternatives in special cases or where resistance to antimonials exists [3, 4]. All these compounds present several problems that limit their use, such as severe side effects, induction of parasite resistance, in-patient administration, and high cost [5]. The novel drug Miltefosine is an effective treatment for visceral leishmaniasis in India but has shown limited efficiency in other countries and for other leishmaniasis forms, besides being teratogenic [4, 6]. All these facts reinforce the need to develop new approaches for leishmaniasis therapy. There are about 28 species classified in the genus *Copaifera*, commonly known as “copaiba,” “copaiva” or “pau-de-óleo” and 16 of these species are endemic in the Brazilian Amazon rainforest and in the Cerrado region [7]. As collected from the trunks of diverse species, copaiba oil is widely employed in folk medicine and cosmetics [7, 8], as an antiinflammatory [9], analgesic agent, [10], antitumoral [11], antimicrobial [12], acaricide [13], larvicide [14], gastro-protector [15, 16], and antinociceptive [17]. The exclusive terpenoid composition of copaiba oils is evidenced by around 72 different types of sesquiterpenes and diterpenes already identified in 27 different species of copaiba oils [7]. Kaurenoic, kovalenic, hardwickiic, polyalthic, and copalic acids are the major diterpenes, while *β*-caryophyllene, *α*-copaene, zingiberene, *β*-bisabolene, and bergamotene are the major sesquiterpenes reported in the oils.

It was demonstrated that the essential oil from *Copaifera reticulata* inhibits the growth of both promastigotes and intracellular amastigotes of *Leishmania amazonensis* [18]. Previous studies showed that oral treatment with *Copaifera martii* oil reduces the average lesion size of a mouse footpad infected by *L. amazonensis* to a degree similar to Glucantime administered by the intramuscular route [19].

In the present study, four chemically standardized commercial copaiba oils were assayed. These oils typify the products that are sold out as popular remedies, in the Amazonian regional markets. They result from the mixing oils, collected from diverse species growing in the extraction area, without prior botanic identification. We performed a bioguided investigation on the antileishmanial ability of these four different commercial batches of copaiba oils, their most active fractions, and *β*-caryophyllene, its more abundant sesquiterpene. The study aims to relate activity to chemical composition rather than botanical identification, in defining therapeutic targets for copaiba oil.

## 2. Material and Methods

### 2.1. Parasites

*L. amazonensis* (WHOM/BR/75/Josefa) promastigotes were cultured at 26°C in Schneider's Insect Medium containing 10% of fetal calf serum (FCS-Crypion), 10% of human urine, and gentamicin (Schering-Plough, RJ, Brazil).

### 2.2. Reagents

Commercial trans-*β*-caryophyllene 99% (up to 1% caryophyllene oxide) was purchased from Sigma-Aldrich (USA). Diazomethane was prepared in ethyl ether from Diazald Aldrich (USA). Commercial samples of copaiba oils C1, C2, and C3 were donated by a cooperative from Acre state (Brazil) and were collected between 1999 and 2000. Samples C1, C2 and C3 originated from the Tarauacá region situated in Acre. Sample C4 was collected in Baía do Portel, PA, Brazil, and donated by an oil extractive company. After receipt, all the samples were transferred and kept in amber glass bottles at 20–22°C. The chemical profiles of the samples were assessed periodically by gas chromatography and shown to remain unchanged. The chemical composition of these commercial oils is given in Tables 1 and 2.

### 2.3. Copaiba Oil Partitioning and Derivatization

Each of the four samples of commercial oils (about 100 g) was submitted to a 2-day exhaustive hydrodistillation (500 mL of distilled water, 8 h) using a modified Clevenger apparatus [20] to separate a colorless volatile fraction and a viscous residue. This latter was decanted from the water and allowed to dry at room temperature. The fractions were termed as volatile fraction (VF), which contains exclusively sesquiterpenes, and nonvolatile fraction (NVF), which is composed exclusively of diterpene acids. Aliquots (10–20 mg) of each original oil and the resinous fractions were dissolved in dichloromethane (2 mL) and treated with diazomethane. The methylated samples and the volatile fractions were then analyzed by gas chromatography.

### 2.4. Gas Chromatography Analysis

Identification of copaiba oil constituent: all oil samples were analyzed by gas chromatography coupled to mass spectrometry (GC-MS), using an HP 6890N equipment fitted with an HP-5 MS capillary column (30 m × 0.32 mm × 0.25 *μ*m film thickness) and processed using MSD Productivity ChemStation Software. The chromatographic conditions were the same as above. The mass analyzer operated at an ion source temperature of 280°C, electron impact ionization energy of 70 eV, and an acquisition mass range from 40 to 500 m/z (3.66 scan/sec). Individual sesquiterpene constituents in the oils were identified by calculating their GC retention indices with reference to a homologous series of normal C10–C30 alkenes and comparing their fragmentation patterns in the mass spectra with those from the Wiley Library Software 59943B and data from the literature [21]. Individual diterpenes were identified by comparing their mass fragmentation with data from the electronic library and published data elsewhere.

### 2.5. Quantitative GC Analysis

Gas chromatography with flame ionization detector (GC-FID) analysis was performed on a gas chromatograph (HP 6890N Network CG System) fitted with a 30 m × 0.32 mm × 0.25 *μ*m film thickness HP-5 capillary column operating in split mode at a ratio of 1 : 50 (split/split less injector). Helium was used as carrier gas (flow 2.5 mL/min; inlet pressure 26.06 psi). The initial oven temperature was kept at 110°C during 2 min, raised to 140°C at 5°C/min and then to 290°C at 20°C/min, remaining at this final level for 10 min. The sample injection volume was 1 *μ*L from a 3 mg/mL solution in CH_2_Cl_2_ and the detector worked at 270°C. The relative abundance of the constituents in the oils was obtained from electronic integration of the signals in the GC-FID chromatograms. Sample injections were carried out in triplicate and standard deviations were considered.

### 2.6. Antipromastigote Activity

Promastigotes (10^6^/mL) were incubated in the presence or absence of different compounds, which were added only once to the cultures. *Leishmania* survival was estimated during five days after exposure to the compounds by counting viable parasites in a Neubauer chamber. Parasites cultured in medium alone were used as a control, and results were expressed as the number of viable parasites for each treatment condition as previously described [22].

### 2.7. Antiamastigote Activity

The antiamastigote activity was measured according to [22]. Briefly, murine peritoneal macrophages were obtained from BALB/c mice, which had been intraperitoneally injected with 3% thioglycolate. After 4 days, stimulated peritoneal macrophages were harvested in RPMI, plated on coverslips, and allowed to adhere for 2 h at 37°C in 5% CO_2_. Nonadherent cells were removed by washing, and adhered macrophages were maintained overnight in RPMI supplemented with 10% FCS, at 37°C, 5% CO_2_. The macrophages were then infected with *L. amazonensis* promastigotes (stationary growth phase) at a 10 : 1 parasite/macrophage ratio and incubated for 1 h at 35°C, 5% CO_2_. Free parasites were washed out with PBS, and cultures were maintained for 24 h at 35°C in 5% CO_2_ in RPMI supplemented with 10% FCS. The oil samples were diluted in dimethyl sulfoxide (DMSO, Sigma) which was used as a control at 0.5%. Different concentrations of the compounds were added to the infected macrophages and, after 24 h of incubation as above, cells were washed, fixed, and stained with Giemsa. The number of amastigotes and the percentage of infected macrophages were determined by counting at least 200 cells in triplicate cultures. Infectivity index was obtained by multiplying the percentage of infected macrophages by the mean number of amastigotes per infected macrophage. Experiments were made in accordance with ethical guidelines for care and handling of laboratory animals.

### 2.8. Cytotoxicity Assays

Murine peritoneal macrophages obtained as above were treated with different concentrations of the compounds for 24 h at 37°C, 5% CO_2_. Macrophages were then washed with PBS, incubated with 0.03% Trypan blue solution, and scored for viable cells. The effect of the compounds on macrophage viability was also determined by the reduction of 2,3-bis[2-methoxy-4-nitro-5-sulfophenyl]-2H-tetrazolium-5-carboxinilide inner salt (XTT, Sigma) assay, as described by [23].

### 2.9. Nitric Oxide Production

Thioglycolate-stimulated mouse peritoneal macrophages obtained as above (5 × 105 cells/well in 24-well plates) were cultured with different concentrations of the compounds concomitant with 100 ng/mL of an IFN-*γ* rich supernatant (4-day culture supernatant of L1210 cell line transfected with IFN-*γ* gene) or 100 ng/mL of lipopolysaccharide (LPS, *E. coli* O111:B4, Sigma) as described by [24]. After 24 h at 37°C in 5% CO_2_, nitrite concentration in culture supernatants was determined by the Griess method [25]. The reaction was read at 540 nm, and the concentration of nitrite was determined with reference to a standard curve using sodium nitrite. Results are expressed as *μ*M concentrations of nitrite.

### 2.10. Nitric Oxide-Trapping Capacity

A cell-free system using an NO donor and VF or NVF was used to test the capacity of these fractions to trap nitric oxide. S-Nitroso-N-acetyl DL-penicillamine (SNAP, Sigma) in solution liberates nitric oxide, which is transformed to nitrite in the medium. The addition of an NO scavenger to the SNAP solution results in a nitrite decay in the supernatant. Using this protocol, VF or NVF (10 *μ*g/mL) was incubated with 1 mM of SNAP. A positive control group was made using rutin, a known NO scavenger (at 1 mM, Sigma). After 6 h of incubation, an aliquot of supernatant was removed to quantify nitrite by the Griess reaction [26]. Results are expressed as *μ*M of nitrite calculated in comparison with the sodium nitrite standard curve.

### 2.11. Statistical Analysis

Data were analyzed by Student's *t*-test when comparing two groups or one-way ANOVA for more than two groups, using the GraphPad 5 Program. *P* values of less than 0.05 were considered significant.

## 3. Results

**Figure 1:**
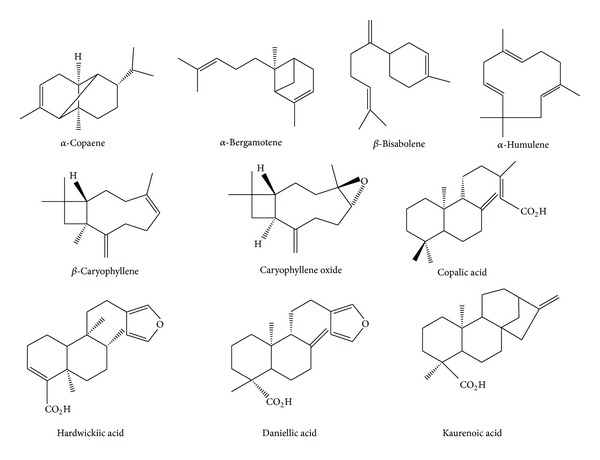
Structure of representative terpenes in the copaiba oil samples.

GC analysis showed C1 and C4 to contain approximately 44.2% and 36.5%  *β*-caryophyllene, respectively; contrary to that observed for samples C2 and C3 which exhibited 12.8% and 5.5%, respectively (Table 1). Only C1 has a significant amount of caryophyllene oxide (10.1%), which is a trace constituent in C2 and C3, and is absent in C4. Additionally, C1 and C4 have a limited amount of *α*-humulene (syn. *α*-caryophyllene), 7.3% and 5.3%, respectively. C4 contained 7.3%  *α*-bergamotene. C3 has 18.1% of *α*-copaene, approximately 7.8% of selinenes and similar amounts (4.5%-4.6%) of *β*-bisabolene and *γ*-cadinene. C2 showed a 12.7% content of *β*-bisabolene (Table 1). Regarding diterpenes, it was observed that eperuic and copalic acids were present in all samples, the latter being more abundant in C1 (6.3%) and C4 (7.6%). Both these oils also have up to 6% of hydroxy and acetoxy derivatives of copalic acid. The oil C2 is characterized by a high content of daniellic acid (33.6%), and C3 is characterized by 10.1% and 9.0% of kaurenoic and hardwickiic acids (Table 1). Hydrodistillation of C4 produced the volatile fraction (VF) containing only sesquiterpenes and the nonvolatile fraction (NVF), consisting of a diterpene acids mixture. A high level of *β*-caryophyllene (42.3%) was found in VF along with 7.5% of oxidized caryophyllenes. Also, as expected, the NVF fraction had a higher copalic acid content (49.9%). The structures of the main constituents in the samples assayed in our study are displayed in Figure 1.

**Figure 2:**
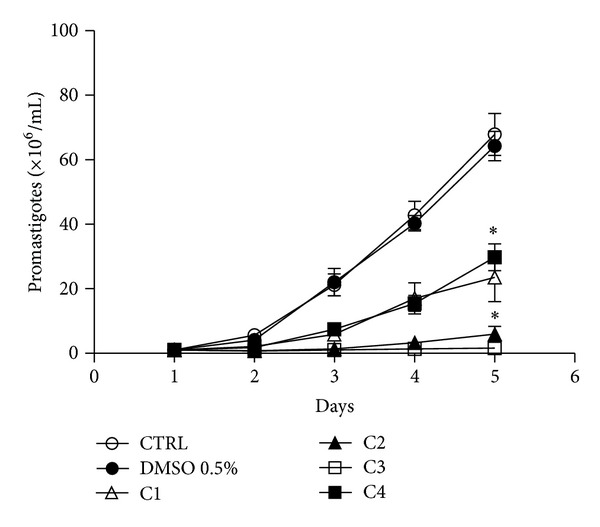
Antipromastigote activity of copaiba oils. *Leishmania amazonensis* promastigotes were treated once with different compounds at 50 *μ*g/mL or with DMSO 0.5% (vehicle). The antipromastigote effect was measured by counting of viable parasites for five days. Results from five experiments are shown as the number of promastigotes ± SEM. *P < 0.01.

**Figure 3:**
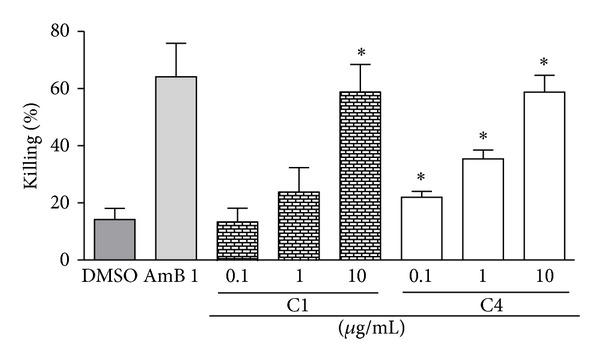
Anti-*Leishmania amazonensis *amastigote activity of C1 and C4. Infected mouse peritoneal macrophages were treated for 24 h with the indicated concentrations of C1 and C4 oils or 1 *μ*g/mL AMB (amphotericin B), or 0.5% of DMSO (vehicle). Results from five experiments in triplicates are shown as % killing ± SEM in relation to the untreated control. *P > 0.05 in relation to DMSO.

The four original copaiba oils, the two fractions distillated from C4 (VF and NFV). and commercial trans-*β*-caryophyllene (CAR) were evaluated for anti-*Leishmania amazonensis* activity. The diterpene-rich C2 and C3 were the more active oils against *L. amazonensis* promastigotes, inhibiting growth by up to 91.3% and 97.5%, respectively, after five-day treatment. The sesquiterpene-rich oils C1 and C4 inhibited, respectively, 65% and 56% promastigote growth (Figure 2). For the evaluation of the antiamastigote properties of the commercial oils, peritoneal macrophages from BALB/c mice infected with *L. amazonensis* were treated for 24 h with 1 *μ*g/mL of C1, C2, C3, and C4 oils and *β*-caryophyllene. Thus C1, C4, and *β*-caryophyllene were selected for further experiments as they presented the highest antiamastigote activity (data not shown). The sesquiterpene-rich C1 and C4 were effective against intracellular amastigotes, in a dose-dependent way with IC_50_ 2.9 *μ*g/mL and IC_50_ 2.3 *μ*g/mL, respectively (Figure 3). These values were, respectively, 6.9- and 8.7-fold more active than that observed for *C. reticulata* crude oil (IC_50_ 20 *μ*g/mL) [18].

**Figure 4:**
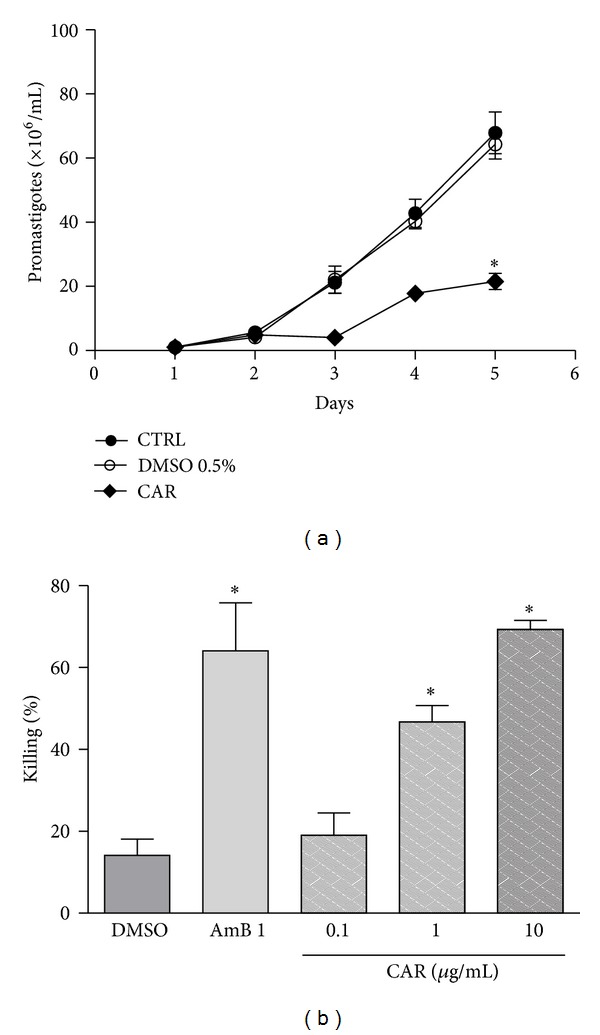
Antileishmanial activity of *β*-caryophyllene (CAR). (a) *Leishmania amazonensis* promastigotes were treated with CAR at 50 *μ*g/mL or with DMSO 0.5% (vehicle). The antipromastigote effect was measured by counting of viable parasites for five days. Results from two experiments are shown as the number of promastigotes ± SEM. *P < 0.01. (b) Mouse peritoneal macrophages infected with *Leishmania amazonensis* amastigote were treated for 24 h with the indicated concentrations of CAR or 1 *μ*g/mL AMB (amphotericin B), or 0.5% of DMSO (vehicle). Results from five experiments in triplicates are shown as % killing ± SEM in relation to the untreated control. *P > 0.05 in relation to DMSO.

Both C1 and C4 are not only the oils with the highest abundance of overall sesquiterpenes but also contained *β*-caryophyllene as their major constituent. This was three- to fourfold higher than that in the oil reported by [18]. In the antipromastigote assay, a 68.3% parasite growth inhibition was obtained after incubation with commercial *β*-caryophyllene (Figure 4(a)), similar to the inhibition by C1 (65%) and C4 (56%) after a five-day treatment (Figure 2). The antiamastigote activity of commercial *β*-caryophyllene against intracellular parasites (Figure 4(b)) was higher than that produced by the oils C1 and C4 (Figure 2). Importantly, *β*-caryophyllene, C1, and C4 at 10 *μ*g/mL exhibited an antiamastigote activity similar to that of amphotericin B at 1 *μ*g/mL, *β*-caryophyllene being slightly more active than the oils at 1 and 10 *μ*g/mL. *β*-Caryophyllene showed a dose-dependent antiamastigote activity, with an IC_50_ of 1.3 *μ*g/mL (6.4 *μ*M). It is also noteworthy that both oils C1 and C4 had the highest *β*-caryophyllene content (Table 1), suggesting that this compound may influence directly the Antileishmanial activity.

**Figure 5:**
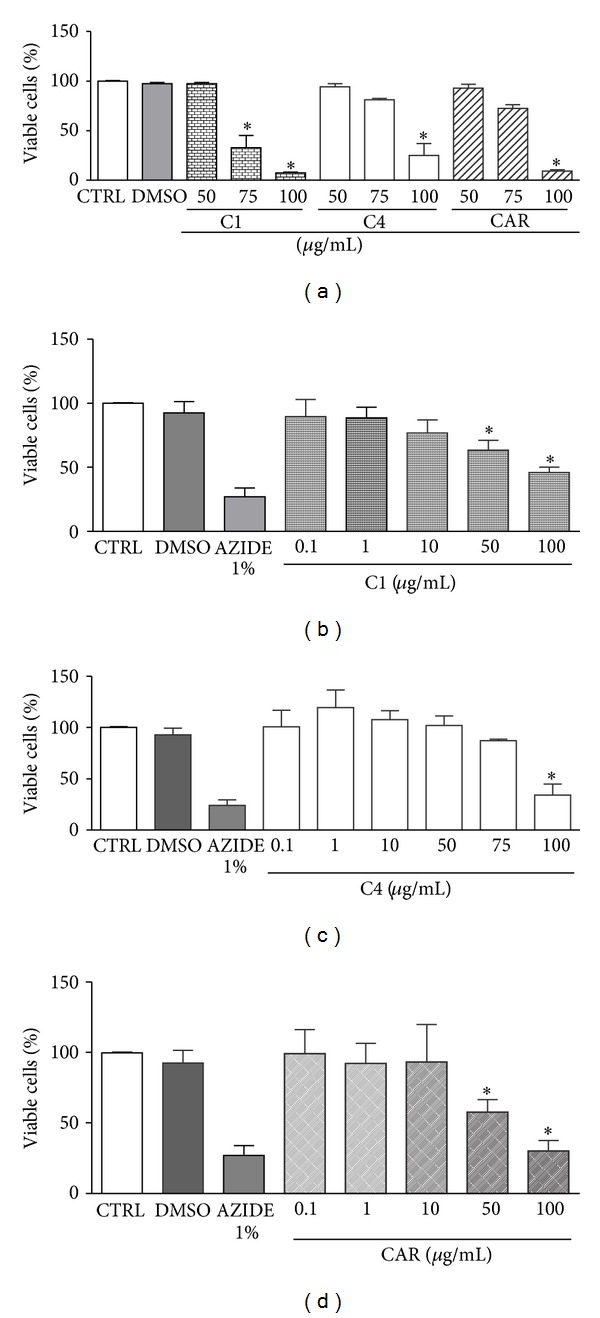
Cytotoxicity of C1, C4 *Copaiba* oils, and *β*-caryophyllene (CAR). Murine peritoneal macrophages were cultured with the compounds at 10 *μ*g/mL for 24 h. Cell viability was verified by Trypan blue dye exclusion assay (a) and XTT method for oil C1 (b), C4 (c), and CAR (d). Cytotoxic activity of 0.5% DMSO (vehicle) and 1% azide were also evaluated. Data represents mean ± SEM of three experiments in duplicates. *P < 0.05 for C1, C4, and CAR versus control.

In order to assess C1, C4, and *β*-caryophyllene cytotoxicity for host cells, membrane integrity was evaluated by the Trypan blue dye exclusion assay carried out in macrophage host cells. Compared to the untreated control, cells remained viable after treatment with C1, C4 or *β*-caryophyllene at dosages up to 50 *μ*g/mL (Figure 5(a)). However, 100 *μ*g/mL of C1, C4, or *β*-caryophyllene caused a significant reduction in cell viability. C1 was the most toxic among them, reducing macrophage viability by 67% at 75 *μ*g/mL and 92.6% at 100 *μ*g/mL. Treatment with C4 reduces the number of viable cells in 18.6% at 75 *μ*g/mL and 74.8% at 100 *μ*g/mL. Cells treated with *β*-caryophyllene showed a decrease of 27.6% and 90.7% in cell viability at the concentrations of 75 *μ*g/mL and 100 *μ*g/mL, respectively. DMSO (0.5%), used to dilute the commercial oils and *β*-caryophyllene, did not harm the membrane of macrophages, indicating that the cell damage was indeed due to the action of the tested substances. 

**Figure 6:**
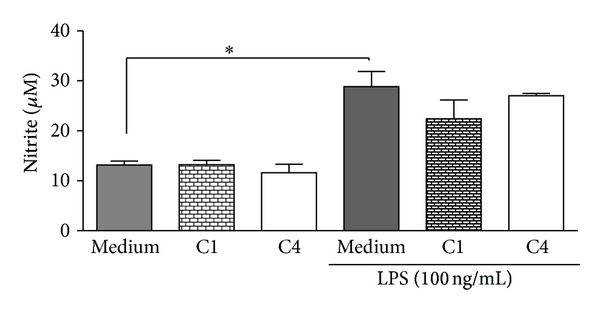
Role of copaiba oils in nitric oxide (NO) production by macrophages. Murine peritoneal macrophages stimulated or not with LPS were treated or not with C1 or C4 at 10 *μ*g/mL. After 48 h the level of NO was estimated by the Griess method. Medium represents untreated and unstimulated and LPS-stimulated macrophages controls. Data represents means ± SEM of three experiments in triplicates. *P < 0.01.

Cytotoxicity of the samples was also assessed by measuring the mitochondrial dehydrogenase activity of the host cell using the XTT method. The results demonstrated that C1, C4, and *β*-caryophyllene at 0.1, 1.0, and 10 *μ*g/mL did not affect cell viability (Figures 5(b), 5(c), and 5(d)). However, a significant reduction of 36.5% and 42.3% in macrophage viability was observed for C1 (Figure 5(b)) and *β*-caryophyllene (Figure 5(d)), respectively, after treating the cells with 50 *μ*g/mL. The C4 oil reduced the viability of the host cells by 12.8% at 75 *μ*g/mL (Figure 5(c)). Among *β*-caryophyllene, C1, and C4, the latter showed lower toxicity to the host cells. The toxic concentration for 50% of macrophages (CC_50_) was 85 *μ*g/mL for C1, 92.4 *μ*g/mL for C4, and 63.6 *μ*g/mL for *β*-caryophyllene. The toxicity to macrophages and the activity against protozoa were compared by using the selectivity index (SI), and its value for amastigotes showed that *β*-caryophyllene is 48.9 times less toxic to the macrophages than to the intracellular parasites. Comparatively, oils C1 and C4 exhibited SI of 29.3 and 40.1, demonstrating a better relationship between efficacy toward amastigotes and toxicity to mammalian cells (Table 2). Nitric oxide (NO) production by macrophages is an important mechanism against intracellular amastigotes. The capacity of murine macrophages to produce NO by treatment with C1 and C4 was then evaluated by using the Griess method. The NO production by macrophages treated with 10 *μ*g/mL of C1 and C4 was similar to that of untreated macrophages. As expected, LPS-stimulated macrophages have an increased NO production in relation to untreated macrophages. Besides, addition of C1 and C4 at 10 *μ*g/mL did not affect the NO production elicited by LPS. These results demonstrate that the leishmanicidal activity of C1 and C4 is independent of the NO production (Figure 6).

**Figure 7:**
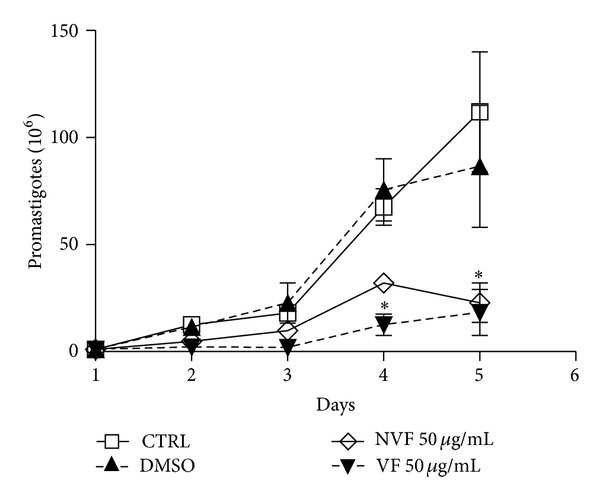
Anti-*Leishmania amazonensis* promastigotes effect of volatile (VF), nonvolatile (NVF) fractions. Promastigotes (1 × 10^6^/mL) were cultured with VF or VNF at 50 *μ*g/mL and DMSO at 0.5%. Results from two experiments in duplicates are shown as the number of promastigotes ± SEM. *P < 0.01.

**Figure 8:**
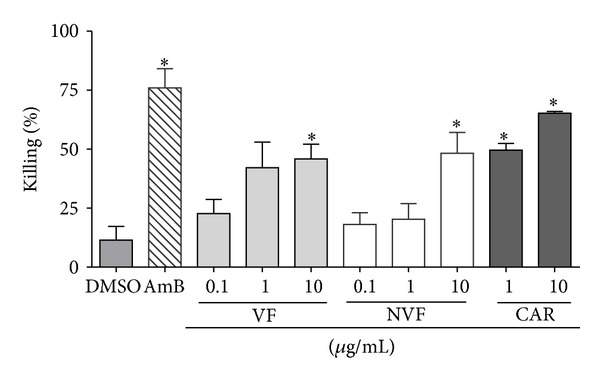
Antiamastigote activity of C4 oil, volatile (VF), nonvolatile (NVF) fractions, and *β*-caryophyllene (CAR). Murine peritoneal macrophages were infected with *Leishmania amazonensis* for 24 h and then treated with the indicated concentrations of the compounds. Amphotericin B (AmB) at 1 *μ*g/mL and 0.5% DMSO (vehicle) were evaluated as controls. Results from two experiments in triplicates are shown as % killing ± SEM in relation, the untreated control. *P < 0.001 relative to DMSO.

Because of its higher antiamastigote activity and lower cytotoxicity for macrophages, C4 was chosen for further fractioning in volatile (VF) and nonvolatile fractions (NVF), which were then assayed for the leishmanicidal effect. Our data show that both VF and NVF at 50 *μ*g/mL inhibited around 80% promastigote growth (Figure 7). Similarly to the C4 oil inhibition profile, a dose-dependent leishmanicidal activity was observed after VF treatment at 0.1, 1.0, and 10 *μ*g/mL, inhibiting amastigote growth by 22.7%, 32.2%, and 50.3%, respectively. At the doses of 1 and 10 *μ*g/mL, *β*-caryophyllene was more effective than both VF and NVF, by inhibiting 52.4 and 64.5% the amastigote growth, respectively. VF and NVF inhibited, respectively, 50.3% and 48.0% the amastigote growth at the higher concentration tested (Figure 8).

**Figure 9:**
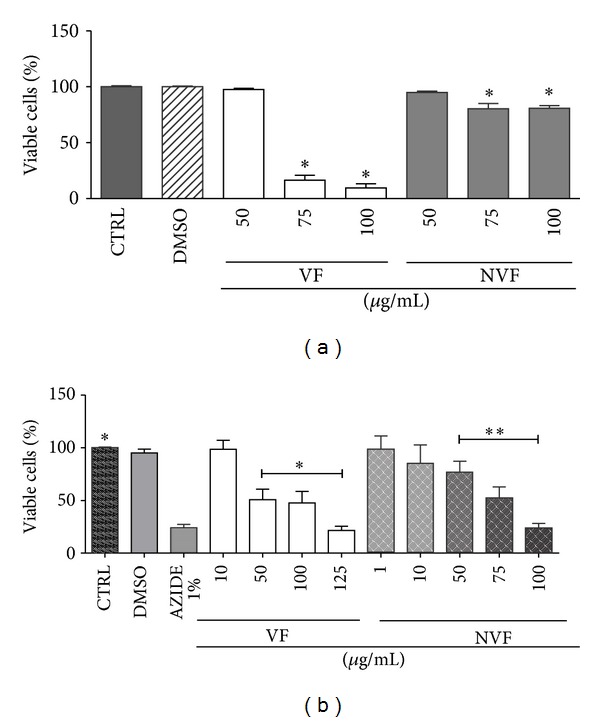
Cytotoxicity of C4 oil, volatile (VF), and nonvolatile (NVF) fractions. Murine peritoneal macrophages were incubated with the indicated concentrations of VF or NVF, and cytotoxicity tested by Trypan blue exclusion (a) and XTT (b) assays. 0.5% DMSO was assayed as the vehicle control and azide at 1% as a positive control for the XTT assay. Results represent the mean ± SEM of five experiments in triplicates. *P < 0.05 and **P = 0.001.

The cytotoxicity of NVF and VF for host cells was also assessed by the Trypan blue exclusion and XTT assays (Figures 9(a) and 9(b)). VF and NVF at 50 *μ*g/mL were not toxic as measured by Trypan blue (Figure 9(a)). The XTT data revealed some mitochondrial toxicity. NVF was less toxic than VF as demonstrated by respective CC_50_s of 77.5 *μ*g/mL and 65.5 *μ*g/mL (Figure 9(b)).

**Figure 10:**
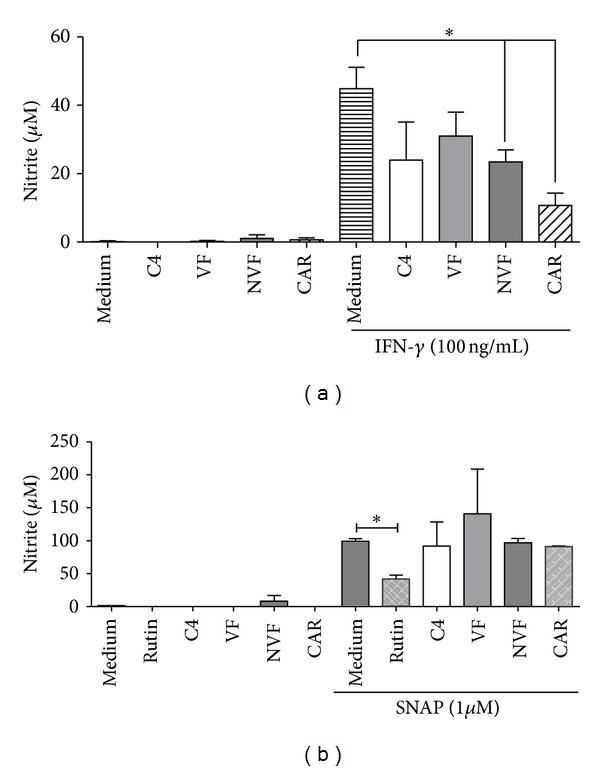
Role of C4 oil volatile (VF) and nonvolatile (NVF) fractions and *β*-caryophyllene (CAR) in nitric oxide (NO) production. (a). Murine peritoneal macrophages activated or not with IFN-*γ* were treated with 10 *μ*g/mL of VF, NVF, and CAR. After 48 h, culture supernatant was collected and nitrite production measured by the Griess method. Medium represents untreated and unstimulated and IFN-*γ*-stimulated macrophages controls. Results represent the mean ± standard error of mean (SEM) of three independent experiments in triplicates. (b). Scavenger activity of VF, NVF, and CAR. Compounds at 10 *μ*g/mL were incubated with 1 *μ*M of SNAP, for 6 h and nitrite production measured by the Griess method. RPMI medium was used as negative control (medium); SNAP as NO donor was incubated with rutin (an NO scavenger) as a control. Results represent the mean ± SEM of three independent experiments. *P < 0.01.

To verify whether the VF, NVF, and *β*-caryophyllene antiamastigote activity was due to NO production by macrophages, cells were treated with C4 fractions at 10 *μ*g/mL and the levels of NO were measured by the Greiss method. Our results revealed that VF and NVF as well as C4 and *β*-caryophyllene did not induce NO production in nonstimulated cells (Figure 10(a)). Furthermore, activation of macrophages by IFN-*γ* stimulates NO production, and NVF, and *β*-caryophyllene at 10 *μ*g/mL reduced IFN-*γ*-induced NO production (Figure 10(a)). Therefore a cell-free assay using SNAP as an NO donor in the presence or absence of C4, VF, NVF and *β*-caryophyllene was performed to confirm the correlation between this inhibition and NO scavenger effect by the samples (Figure 10(b)). Rutin (an NO trapping substance) added to the SNAP solution reduced the NO levels by 70%, while the addition of VF, NVF, C4, and *β*-caryophyllene at 10 *μ*g/mL to SNAP did not reduce the NO levels (Figure 10(b)), indicating that NO decrease was not due to scavenging.

## 4. Discussion

The effort to associate diverse biological activity with botanically certified species of *Copaifera *has been expended, as recently reviewed by [27]. However, the constitution of copaiba oil varies widely either quantitatively or qualitatively, both in regard to the overall phytochemical profile and to the percentage content of individual chemical markers. Physical characteristics of the soil, tree diameter, time of year, and other factors can affect the characteristics of the oil. Distinct compositions of oils from the same species, depending on the local of collection, have already been reported for *C. reticulata* [28], *C. guianensis*, *C. duckei*, and *C. multijuga* [28, 29]. Furthermore, the bulk oil provided by local collectors is usually made up of mixtures coming from a variety of different trees, without any botanical control. Also important is the variation of sesquiterpenes and diterpene acid content in oils from different botanical origins. Controlling all these variables in a phytocomplex to guarantee its chemical reproducibility and therapeutic effectiveness seems difficult to achieve in the case of copaiba oil, especially in large-scale production where even botanical certification is not a valid tool to control the raw material's chemical composition.

For this reason, in the present study, we opted for a bioguided-type antileishmanial assay applied to commercial oil mixtures without attention to botanical issues. Bulk mixtures of copaiba oils classified according to the areas of their collection in the Amazon region, just as supplied to the popular markets as folk remedies, were chosen for study. The primary goal included the identification of potential chemical markers and the standardization of enriched fractions from the original oil mixtures. This experimental approach permitted the identification of *β*-caryophyllene as an antileishmanial compound in the most active oil fractions, the relative content of this compound being the standard marker for leishmaniasis treatment.

Santos and colleagues [18] assessed the leishmanicidal activity of oleoresins obtained from nine botanically certified *Copaiba* species and showed that *C. reticulata* oil was the most active sample against *L. amazonensis* promastigotes and amastigotes as well as the one with the lowest toxicity to the host cells. This species has *β*-caryophyllene as the predominant constituent (40.9% in 78.2% total sesquiterpenes in the sample studied), a result in accord with the definition of this compound as a primary effective marker for the development of antileishmanial phytomedicines from copaiba oil. *C. reticulata *oleoresin was also active against *L. chagasi *amastigotes and promastigotes [30].

*β*-Caryophyllene is a lipophilic volatile sesquiterpene present in essential oils of various plants; it is commonly ingested with plant foods and used as an additive in cosmetics [31]. The compound presents antimicrobial, antioxidant, anesthetic, and anti-inflammatory activities as well as exhibiting anticancer potential through its toxic action towards several tumor cell lines [31–35]. To this extensive list of activities of *β*-caryophyllene, its antileishmanial property can now be added although the mechanism of action is presently unknown. Our results have shown that nitric oxide production, which is a potent leishmanicidal mechanism in macrophages, was not induced by *β*-caryophyllene. Moreover, we demonstrated that *β*-caryophyllene inhibited nitric oxide production induced by IFN-*γ* in macrophages, as already reported [36]. The nitric oxide inhibitory effect could have been mediated by the agonist action of *β*-caryophyllene on the cannabinoid type 2 receptor (CB_2_) expressed by macrophages, which has been shown to inhibit LPS-induced inflammatory response [37]. Importantly, the higher selectivity for amastigotes compared with host cells is important, but further studies are needed to better characterize the leishmanicidal mechanism. Interestingly, Santos and coworkers [38] reported that *β*-caryophyllene was not active against *Leishmania *axenic amastigotes. This discrepancy from the present observations with intramacrophage amastigotes could be due to differences in the parasite tested forms. The intramacrophage parasite better mimics the natural infection than axenic amastigotes. It could be that *β*-caryophyllene is metabolized by the macrophages to an active molecule with leishmanicidal activity. This assumption is reinforced by *β*-caryophyllene antitrypanosomal activity against intracellular *Trypanosoma cruzi* amastigotes [39].

Cytotoxicity determination of copaiba oil—and essential oils in general—is not simple. Data on toxic effects reported for individual terpenoids are scarce and synergistic effects are probablly present in the complex mixtures. Loizzo and coauthors [40] stated that the correlation usually found between the major compounds in essential oils and observed pharmacological effects is generally not extended to cytotoxicity. Minor compounds may be primarily responsible for cytotoxic effects. The cytotoxicity of other sesquiterpenes commonly present in copaiba oils may be inferred from the few studies involving the effect of essential oil constituents on cancer cells. For instance, aromadendrene enhances synergistically the efficacy of some well-established chemotherapeutic agents [41], and essential oils containing cadinenes have been demonstrated to inhibit the growth of several human cancer cell lines [42, 43]. Thus, although the higher content of *β*-caryophyllene and its oxide in C1 could be correlated with the antiamastigote effect shown by C1 (IC_50_ 2.9 *μ*g/mL), which showed higher toxicity for macrophages than C4, possibly explainable by the absence of *β*-caryophyllene oxide in C4. This compound is known to interfere with different cellular pathways leading to cell apoptosis [44].

Concerning the leishmanicidal activity, fractionation of C4 enhanced promastigote killing capacity of VF and NVF in comparison to the whole C4 oil. A similar enhancement was not observed for the amastigote killing activity, probably due to the same content of *β*-caryophyllene in C4 and VF. The diterpene-rich fraction (NVF) was active against amastigotes only at the highest concentration tested. However, the cytotoxicity to the host cells was higher for VF than for C4 oil. This could be explained by the presence of *β*-caryophyllene oxide in VF (formed during C4 distillation), already reported to be toxic, causing mitochondrial cell damage leading to apoptosis [44].

Regarding the diterpene acids, attention is drawn to their significant content in furanoid derivatives (daniellic and hardwickiic acids) in C1 and C3 oils. Furans are known to be relatively labile heteroaromatic compounds, especially toward electrophilic substrates. Furan itself is known to be oxidized by cytochrome P450 to yield chemically reactive and cytotoxic *α*, *β*-unsaturated dialdehydes [45]. However, oral administration to Wistar rats of *Copaifera duckei* oil (containing 59.3% of furanoid hardwickiic acid), for three consecutive days, only showed significant cytotoxicity at doses between 25% and 50% of the previously assessed LD_50_ dose [46]. Interestingly, C2 and C3 oils presented 34.7% of furanoid derivatives, which were absent in C1 e C4.

Importantly, the absence of genotoxicity and mutagenicity to mice, administered with acute single doses up to 2 g/Kg of copaiba oil or its volatile and nonvolatile fractions, has been demonstrated using the comet assay as well as by the absence of any increase in the frequency of micronuclei of bone marrow and peripheral blood cells [47]. Moreover, oral treatment with whole *C. martii *oil reduces *L. amazonensis* lesions in mice [19]. 

Taken together, the present findings suggest that *β*-caryophyllene could constitute an attractive and safe molecule for development of an antileishmanial drug as indicated in the present study. It also emerges as a recommendable marker for copaiba oils. Moreover, oils standardized for their *β*-caryophyllene content could provide an affordable treatment for leishmaniasis, in endemic areas.

## Figures and Tables

**Table 1:** Constituents of the commercial copaiba oils.

Constituent		Relative abundance (%)^1^					
	RI^2^	C1	C2	C3	C4	C4-VF	C4-NVF
Sesquiterpenes							
*δ*-Elemene	1340				0.87 ± 0.05	1.26 ± 0.14	
*α*-Cubebene	1350			2.41 ± 0.02	0.72 ± 0.07	0.89 ± 0.15	
*α*-Copaene	1379	1.57 ± 0.12		**18.16 ± 0.15**	7.18 ± 0.26	11.22 ± 0.73	
*β*-Elemene	1391	0.79 ± 0.08		2.39 ± 0.02	1.47 ± 0.00	1.96 ± 0.30	
*β*-Caryophyllene (*trans*)	1427	**44.23 **± **3.20**	12.83 ± 0.13	5.53 ± 0.04	**36.46 ± 1.69**	**42.36 ± 5.66**	
*α*-Bergamotene	1439	0.97 ± 0.06	**11.84 ± 0.15**	1.99 ± 0.02	7.29 ± 0.33	**9.11 ± 1.26**	
*β*-Humulene	1453				1.18 ± 0.02	1.45 ± 1.19	
*α*-Humulene	1465	**7.29 ± 0.49**	2.43 ± 0.08	1.10 ± 0.01	5.30 ± 0.06	5.63 ± 1.10	
Allo-aromadendrene	1469			1.16 ± 0.02	0.66 ± 0.07	0.76 ± 0.38	
*γ*-Muurolene	1483				2.49 ± 0.03	2.37 ± 0.35	
Germacrene D	1488				4.56 ± 0.013	2.65 ± 0.55	
*β*-Selinene	1493	0.46 ± 0.02	2.40 ± 0.03	4.74 ± 0.03	0.62 ± 0.01	—	
*α*-Selinene	1499	0.35 ± 0.03	1.46 ± 0.01	3.03 ± 0.05			
Bicyclogermacrene	1500				1.00 ± 0.01	0.45 ± 0.14	
*α*-Muurolene	1500				1.04 ± 0.01	0.46 ± 0.36	
*β*-Bisabolene	1509	1.55 ± 0.11	12.65 ± 0.93	4.50 ± 0.02	1.26 ± 0.01	1.04 ± 0.64	
*δ*-Amorphene	1514				5.14 ± 0.16	1.14 ± 0.67	
*β*-Sesquiphellandrene	1522		0.72 ± 0.06				
*γ*-Cadinene	1522	0.80 ± 0.06		4.61 ± 0.01	1.55 ± 0.02	2.01 ± 0.56	
Iso-*γ*-Bisabolene (*E*)	1534		0.88 ± 0.02	0.48 ± 0.01			
Germacrene B	1565	0.67 ± 0.06					
Caryophyllene alcohol	1578	1.00 ± 0.18					
Caryophyllene oxide	1589	**10.17 ± 0.77**	0.85 ± 0.04	1.10 ± 0.02		**6.83 ± 0.62**	
Humulene epoxide II	1608					0.72 ± 0.09	
Unknow [M]^+^ 322	1631				1.31 ± 0.02	0.96 ± 0.18	
*α*-Muurolol	1642	2.12 ± 0.16		0.60 ± 0.01			
*α*-Cadinol	1651				1.55 ± 0.90	0.70 ± 0.12	
Diterpenes^3^							
Unknown acid (kaurene type; [M]^+^ 294)		traces					
Kaur-16-ene		traces		3.08 ± 0.03			
Eperuic acid		0.69 ± 0.13	2.31 ± 0.05	2.61 ± 0.02	0.62 ± 0.13		4.69 ± 0.62
Clerod-3-en-15-oic acid isomer ([M]^+^ 320)			3.13 ± 0.06	5.28 ± 0.04			
Labd-7-en-15-oic acid			1.07 ± 0.04	0.80 ± 0.01			
Kaurenoic acid				**10.10 ± 0.09**			
Copalic acid		**6.32 ± 0.81**	2.82 ± 0.40	2.16 ± 0.02	**7.62 ± 0.62**		**49.87 ± 1.06**
Kovalenic acid		0.43 ± 0.06	1.75 ± 0.32				
Daniellic acid		3.16 ± 0.43	**33.65 ± 1.05**	**8.25 ± 0.19**			
Hardwickiic acid			0.77 ± 0.08	**900 ± 0.07**			
Pinifolic acid		0.51 ± 0.10	3.65 ± 0.19				
Agathic acid		2.66 ± 0.30			0.95 ± 0.31		6.65 ± 0.10
Hydroxy-copalic acid		1.80 ± 0.27			1.20 ± 0.76		9.39 ± 0.14
Acetoxy-copalic acid		4.13 ± 1.21			2.97 ± 0.32		**19.56** ± 0.48

Total sesquiterpenes		**71.96**	**46.06**	**51.81**	**81.65**		
Total diterpenes		**19.71**	**49.15**	**41.28**	**13.36**		
Total identified		**91.67**	**95.21**	**93.09**	**95.01**		

^1^Values for C1–C4 and C4-NVF: from the relative peak area in the chromatograms; for C4-VF: quantification using commercial *β*-caryophyllene as analytical standard. ^2^RI = retention index. See Adams, 2007 [21]. ^3^Analyzed as the methyl ester derivatives. In bold: more abundant constituents in the oil samples.

**Table 2:** Selectivity index (SI) for C1 and C4 copaiba oils and *β*-caryophyllene (CAR).

Compound	CC_50 _(*µ*g/mL)	IC_50 _(*µ*g/mL)	SI
C1	85.0	2.9	29.3
C4	92.4	2.3	40.1
CAR	63.6	1.3	48.9

CC_50_: cytotoxic concentration for 50% macrophages based on the XTT data.

IC_50_: inhibitory concentration for 50% amastigotes.

## References

[1] Desjeux P (2004). Leishmaniasis: current situation and new perspectives. *Comparative Immunology, Microbiology and Infectious Diseases*.

[2] Alvar J, Vélez ID, Bern C (2012). Leishmaniasis worldwide and global estimates of its incidence. *PLoS One*.

[3] Murray HW, Berman JD, Davies CR, Saravia NG (2005). Advances in leishmaniasis. *The Lancet*.

[4] Croft SL, Barrett MP, Urbina JA (2005). Chemotherapy of trypanosomiases and leishmaniasis. *Trends in Parasitology*.

[5] Berman JD (2003). Current treatment approaches to leishmaniasis. *Current Opinion in Infectious Diseases*.

[6] Seifert K, Croft SL (2006). *In vitro* and *in vivo* interactions between miltefosine and other antileishmanial drugs. *Antimicrobial Agents and Chemotherapy*.

[7] Veiga VF, Pinto AC (2002). The *Copaifera* L. genus. *Quimica Nova*.

[8] Wadt LHO, De Jáuregui KMCH, De Araْjo EA, Felinto AS, Vieira AH, Bentes-Gama M Efeito do tipo e época de extração na produção do óleo-resina de Copaíba.

[9] Veiga VF, Rosas EC, Carvalho MV, Henriques MGMO, Pinto AC (2007). Chemical composition and anti-inflammatory activity of copaiba oils from *Copaifera cearensis* Huber ex Ducke, *Copaifera reticulata* Ducke and *Copaifera multijuga* Hayne—a comparative study. *Journal of Ethnopharmacology*.

[10] Carvalho JCT, Cascon V, Possebon LS (2005). Topical antiinflammatory and analgesic activities of *Copaifera duckei* Dwyer. *Phytotherapy Research*.

[11] Lima SRM, Veiga VF, Christo HB, Pinto AC, Fernandes PD (2003). *In vivo* and *in vitro* studies on the anticancer activity of *Copaifera multijuga* Hayne and its fractions. *Phytotherapy Research*.

[12] Dos Santos AO, Ueda-Nakamura T, Dias Filho BP, Veiga VF, Pinto AC, Nakamura CV (2008). Antimicrobial activity of Brazilian copaiba oils obtained from different species of the *Copaifera* genus. *Memorias do Instituto Oswaldo Cruz*.

[13] Fernandes FDF, Freitas EDPS (2007). Acaricidal activity of an oleoresinous extract from *Copaifera reticulata* (Leguminosae: Caesalpinioideae) against larvae of the southern cattle tick, Rhipicephalus (Boophilus) microplus (Acari: Ixodidae). *Veterinary Parasitology*.

[14] Da Silva HHG, Geris R, Rodrigues Filho E, Rocha C, Da Silva IG (2007). Larvicidal activity of oil-resin fractions from the Brazilian medicinal plant *Copaifera* reticulata Ducke (Leguminosae-Caesalpinoideae) against *Aedes aegypti* (Diptera, Culicidae). *Revista da Sociedade Brasileira de Medicina Tropical*.

[15] Paiva LAF, Rao VSN, Gramosa NV, Silveira ER (1998). Gastroprotective effect of *Copaifera* langsdorffii oleo-resin on experimental gastric ulcer models in rats. *Journal of Ethnopharmacology*.

[16] Bento AF, Marcon R, Dutra RC (2011). *β*-caryophyllene inhibits dextran sulfate sodium-induced colitis in mice through CB2 receptor activation and PPAR*γ* pathway. *American Journal of Pathology*.

[17] Gomes NM, Rezende CM, Fontes SP, Matheus ME, Fernandes PD (2007). Antinociceptive activity of Amazonian Copaiba oils. *Journal of Ethnopharmacology*.

[18] Santos AO, Ueda-Nakamura T, Veiga Junior VF, Pinto AC, Nakamura CV (2008). Effect of Brazilian copaiba oils on *Leishmania amazonensis*. *Journal of Ethnopharmacology*.

[19] dos Santos AO, Costa MA, Ueda-Nakamura T (2011). *Leishmania amazonensis*: effects of oral treatment with copaiba oil in mice. *Experimental Parasitology*.

[20] Wasicky R (1963). Essencial oil extractor apparatus. Modification of Clevenger apparatus. *Revista de Farmácia e Bioquímica da Universidade de São Paulo*.

[21] Adams R (2007). *Identification of Essential Oil Components by Gas Chromatography / Mass Spectrometry*.

[22] Ferreira C, Soares DC, Barreto-Junior CB (2011). Leishmanicidal effects of piperine, its derivatives, and analogues on *Leishmania amazonensis*. *Phytochemistry*.

[23] Roehm NW, Rodgers GH, Hatfield SM, Glasebrook AL (1991). An improved colorimetric assay for cell proliferation and viability utilizing the tetrazolium salt XTT. *Journal of Immunological Methods*.

[24] Green SJ, Meltzer MS, Hibbs JB, Nacy CA (1990). Activated macrophages destroy intracellular *Leishmania major* amastigotes by an L-arginine-dependent killing mechanism. *Journal of Immunology*.

[25] Soares DC, Pereira CG, Meireles MÂA, Saraiva EM (2007). Leishmanicidal activity of a supercritical fluid fraction obtained from *Tabernaemontana catharinensis*. *Parasitology International*.

[26] Field L, Dilts RV, Ravichandran R, Lenhert PG, Carnahan GE (1978). An unusually stable thionitrite from N-acetyl-D,L-penicillamine; X-ray crystal and molecular structure of 2-(acetylamino)-2-carboxy-1,1-dimethylethyl thionitrite. *Journal of the Chemical Society, Chemical Communications*.

[27] Leandro LM, De Sousa Vargas F, Barbosa PCS, Neves JKO, Da Silva JA, Da Veiga VF (2012). Chemistry and biological activities of terpenoids from copaiba (*Copaifera* spp.) oleoresins. *Molecules*.

[28] Cascon V, Gilbert B (2000). Characterization of the chemical composition of oleoresins of *Copaifera guianensis* Desf., *Copaifera duckei* Dwyer and *Copaifera multijuga* Hayne. *Phytochemistry*.

[29] Biavatti MW, Dossin D, Deschamps FC, Lima MP (2006). Copaiba oil-resin analysis: contribution to quality control. *Brazilian Journal of Pharmacognsosy*.

[30] Rondon FC, Bevilaqua CM, Accioly MP (2012). *In vitro* efficacy of *Coriandrum sativum*, *Lippia sidoides* and *Copaifera reticulata* against *Leishmania chagasi*. *Revista Brasileira de Parasitologia Veterinaria*.

[31] Ghelardini C, Galeotti N, Di Cesare Mannelli L, Mazzanti G, Bartolini A (2001). Local anaesthetic activity of *β*-caryophyllene. *Farmaco*.

[32] Amiel E, Ofir R, Dudai N, Soloway E, Rabinsky T, Rachmilevitch S (2012). *β*-Caryophyllene, a compound isolated from the biblical balm of gilead (*Commiphora gileadensis*), is a selective apoptosis inducer for tumor cell lines. *Evidence-Based Complementary and Alternative Medicine*.

[33] Sabulal B, Dan M, J AJ (2006). Caryophyllene-rich rhizome oil of *Zingiber nimmonii* from South India: chemical characterization and antimicrobial activity. *Phytochemistry*.

[34] Kubo I, Chaudhuri SK, Kubo Y (1996). Cytotoxic and antioxidative sesquiterpenoids from Heterotheca inuloides. *Planta Medica*.

[35] Ashour ML, El-Readi M, Youns M (2009). Chemical composition and biological activity of the essential oil obtained from *Bupleurum marginatum* (Apiaceae). *Journal of Pharmacy and Pharmacology*.

[36] Tung Y-T, Chua M-T, Wang S-Y, Chang S-T (2008). Anti-inflammation activities of essential oil and its constituents from indigenous cinnamon (*Cinnamomum osmophloeum*) twigs. *Bioresource Technology*.

[37] Gertsch J (2008). Anti-inflammatory cannabinoids in diet: towards a better understanding of CB(2) receptor action?. *Communicative & Integrative Biology*.

[38] Nakamura CV, Dos Santos AO, Ueda-Nakamura T, Dias Filho BP, Da Veiga Junior VF (2012). Copaiba oil: an alternative to development of new drugs against leishmaniasis. *Evidence-Based Complementary and Alternative Medicine*.

[39] Izumi E, Ueda-Nakamura T, Veiga VF, Pinto AC, Nakamura CV (2012). Terpenes from *Copaifera* demonstrated *in vitro* antiparasitic and synergic activity. *Journal of Medicinal Chemistry*.

[40] Loizzo MR, Tundis R, Menichini F, Saab AM, Statti GA, Menichini F (2008). Antiproliferative effects of essential oils and their major constituents in human renal adenocarcinoma and amelanotic melanoma cells. *Cell Proliferation*.

[41] Eid SY, El-Readi MZ, Wink M (2012). Digitonin synergistically enhances the cytotoxicity of plant secondary metabolites in cancer cells. *Phytomedicine*.

[42] Lampronti I, Saab AM, Gambari R (2006). Antiproliferative activity of essential oils derived from plants belonging to the Magnoliophyta division. *International Journal of Oncology*.

[43] Mulyaningsih S, Youns M, El-Readi MZ (2010). Biological activity of the essential oil of *Kadsura longipedunculata* (Schisandraceae) and its major components. *Journal of Pharmacy and Pharmacology*.

[44] Park K-R, Nam D, Yun H-M (2011). *β*-Caryophyllene oxide inhibits growth and induces apoptosis through the suppression of PI3K/AKT/mTOR/S6K1 pathways and ROS-mediated MAPKs activation. *Cancer Letters*.

[45] Esenfeld SM (2011). *Identification of target proteins of furan reactive metabolites in rat liver [Ph.D. thesis]*.

[46] Maistro EL, Tavares Carvalho JC, Cascon V, Coelho Kaplan MA (2005). *In vivo* evaluation of the mutagenic potential and phytochemical characterization of oleoresin from *Copaifera duckei* Dwyer. *Genetics and Molecular Biology*.

[47] Almeida MR, Joana D’Arc Castania Darin JDC, Hernandes LC, Ramos MFS, Antunes LMG, Freitas O (2012). Genotoxicity assessment of Copaiba oil and its fractions in Swiss mice. *General Molecular Biology*.

